# The effects of improving service efficiency in a specialist inpatient eating disorder treatment program

**DOI:** 10.1186/2050-2974-3-S1-O13

**Published:** 2015-11-23

**Authors:** Urvashnee Singh, Kate Fleming, Fiona Cartwright, Fiona Salter

**Affiliations:** 1 The Hollywood Clinic, Australia

## 

The Hollywood Clinic is a private adult eating disorder inpatient service in Western Australia with ten specialised beds. It was identified that this service had a long length of stay, which was shown to limit access to care and made the program less cost-effective. Not only are there sizable financial savings in reduced length of stay but indirect “costs” to patients may include missed school and work, impoverished relationships and decreased quality of life. Conversely, early discharge resulting from pressure on length of stay may negatively impact treatment outcomes and recovery trajectories. Our objective then, was to optimise patients' length of stay.

We introduced a range of initiatives to improve program efficiency. These included earlier increases in oral intake prescription, higher energy content of menus, closer monitoring of weight restoration, stream-lining our protocol and utilising our day patient program on discharge. These initiatives resulted in a reduction in the average length of stay from 52 days to 35 days. Concomitantly, there has been no reduction in treatment gains during shorter admissions. Furthermore, there is currently no waiting list for admission to our inpatient eating disorder program, giving us increased capacity to treat more patients in need of the service.

